# Diversity of *Babesia* spp. in skunks from selected states in the United States of America[Fn FN1]

**DOI:** 10.1051/parasite/2024043

**Published:** 2024-07-24

**Authors:** Kayla B. Garrett, Justin Brown, Mourad Gabriel, Robert Dowler, J. Clint Perkins, Dianna Krejsa, Michael J. Yabsley

**Affiliations:** 1 Southeastern Cooperative Wildlife Disease Study, Veterinary Medicine, University of Georgia 589 D. W. Brooks Dr. Athens GA 30602 USA; 2 Warnell School of Forestry and Natural Resources, University of Georgia 180 E. Green St. Athens GA 30602 USA; 3 Department of Veterinary and Biomedical Sciences, Penn State University 108 AVBS Building University Park PA 16802 USA; 4 Karen C. Drayer Wildlife Health Center, University of California Davis School of Veterinary Medicine Davis CA 95616 USA; 5 Integral Ecology Research Center 239 Railroad Ave Blue Lake CA 95525 USA; 6 Department of Biology, Angelo State University San Angelo TX 76909 USA; 7 Department of Natural Resource Management, Texas Tech University 2903 15th Street Lubbock TX 79409 USA; 8 Center for the Ecology of Infectious Diseases, University of Georgia Athens GA 30602 USA

**Keywords:** Striped skunk, Spotted skunk, Hog-nosed skunk, *Babesia microti*-like species, Piroplasms, Tick-borne pathogens

## Abstract

*Babesia* species are intraerythrocytic protozoan parasites that infect a variety of hosts. The goal of this study was to evaluate the piroplasm species present in skunks in various states in the United States and determine whether there was any geographic variation. Spleen, whole blood, or blood on filter paper were received from Pennsylvania, Kentucky, North Carolina, South Carolina, Georgia, Missouri, Louisiana, Texas, Kansas, and California, and were tested for *Babesia* sp. We tested four species of skunks including striped skunk (*Mephitis mephitis, n* = 72), eastern spotted skunk (*Spilogale putorius, n* = 28), western spotted skunk (*Spilogale gracilis, n* = 15), and hog-nosed skunk (*Conepatus leuconotus, n* = 11). A PCR assay targeting the 18S rRNA region and *cox1* region were used to determine if skunks were infected with piroplasms and for phylogenetic analyses. A total of 48.4% (61/126) of skunks tested positive for a *Babesia* species. Both the 18S and *cox1* analysis supported a skunk-specific *Babesia microti*-like sp. of carnivores as well as a species in the *B. microti* complex that is phylogenetically unique from both *B. microti* of humans and the *B. microti-*like sp. of carnivores. In the 18S analysis, there was a third species of *Babesia* in hog-nosed skunks in the western piroplasm group. This study shows that at least three species of piroplasms occur in skunk species in the United States and further highlights the importance of phylogenetic analyses and the use of multiple gene targets when studying piroplasms.

## Abstract (fr)

Les espèces de *Babesia* sont des protozoaires parasites intraérythrocytaires qui infectent divers hôtes. Le but de cette étude était d’évaluer les espèces de piroplasmes présentes chez les mouffettes dans divers états des États-Unis et de déterminer s’il existait une variation géographique. Des rates, du sang total ou du sang sur papier filtre ont été reçus de Pennsylvanie, du Kentucky, de Caroline du Nord, de Caroline du Sud, de Géorgie, du Missouri, de Louisiane, du Texas, du Kansas et de Californie, et ont été testés pour *Babesia* sp. Nous avons testé quatre espèces de mouffettes, dont la mouffette rayée (*Mephitis mephitis*, *n* = 72), la mouffette tachetée de l’Est (*Spilogale putorius*, *n* = 28), la mouffette tachetée de l’Ouest (*Spilogale gracilis*, *n* = 15) et la mouffette à nez plat (*Conepatus leuconotus, n* = 11). Un test PCR ciblant la région de l’ARNr 18S et la région *cox1* a été utilisé pour déterminer si les mouffettes étaient infectées par des piroplasmes et pour des analyses phylogénétiques. Au total, 48,4 % (61/126) des mouffettes ont été testées positives pour une espèce de *Babesia*. Les analyses du 18S et du *cox1* ont toutes deux confirmé une espèce de type *Babesia microti* de carnivores spécifique aux mouffettes ainsi qu’une espèce du complexe *B. microti* qui est phylogénétiquement unique à la fois par rapport à *B. microti* de l’homme et à l’espèce des carnivores. Dans l’analyse 18S, il y avait une troisième espèce de *Babesia* chez les mouffettes à nez plat du groupe des piroplasmes de l’ouest. Cette étude montre qu’au moins trois espèces de piroplasmes sont présentes chez les espèces de mouffettes aux États-Unis et souligne en outre l’importance des analyses phylogénétiques et de l’utilisation de plusieurs cibles génétiques lors de l’étude des piroplasmes.

## Introduction

Piroplasms are small, intraerythrocytic, protozoan parasites (Order Piroplasmida) represented by numerous genera including the medically and veterinary relevant *Babesia, Theileria,* and *Cytauxzoon*. The genus *Babesia* is polyphyletic and comprised of at least three major clades: the *Babesia sensu stricto* species (true babesiids), *Babesia sensu lato* species (western piroplasms), and the *Babesia microti* and *B. microti*-like species [26]. There is considerable debate on the taxonomy of this group, but it is generally accepted that based on molecular and biological data, the western piroplasms and the *Babesia microti*/*B. microti*-like species should be reclassified into two new genera; however, until that time, new species are being described in these two groups in the genus *Babesia*. Specifically, for the *B. microti*-like group, there have been recent efforts to formally describe species that infect mammals, such as *Babesia vulpes* (=*Theileria annae*) of red fox (*Vulpes vulpes*) and domestic dogs [2, 3]. However new *B. microti*-like sp. in mammals continue to be detected and characterized but are currently formally unnamed, such as the *B. microti-*like species of raccoons (*Procyon lotor*) and a *B. microti-*like sp. of North American river otters (*Lontra canadensis*) [6, 10, 11, 13]. A major problem when attempting to formally describe piroplasms is the disconnect between old descriptions and new data. Many piroplasm species were initially described based only on morphology; however, recent molecular studies have shown that many genetically distinct species are morphologically identical [5]. Examples of this include the species *Babesia lotori* of raccoons being a possible species complex [5, 10].

*Babesia mephitis* Holbrook & Frerichs, 1970 was described from striped skunks (*Mephitis mephitis*) in Maryland, USA; however, no additional work on this parasite has been conducted [16]. This parasite was described based on morphologic features on blood films and was included in the ‘large *Babesia’* group (historically babesiids were loosely classified as large or small based on size; however, genetic phylogenies do not support this classification) [5, 16]. To date, the only other reports of *Babesia* species in striped skunks include a molecular detection of a piroplasm in the *B. microti*-like group in a single skunk from Massachusetts, USA, and a striped skunk from New York, USA with a piroplasm most similar to *B. microti;* however, the sequence for this sample is not available [13, 15]. Skunks in the United States share similar habitats with other species that can be infected with *B. microti*-like sp., such as red foxes and raccoons, and are parasitized by a similar tick community, including: *Ixodes cookei, I. marxi, I. texanus* and *Dermacentor variabilis* [8, 27].

The purpose of this study was to genetically characterize piroplasms in striped skunks, eastern spotted skunks (*Spilogale putorius*), western spotted skunks (*Spilogale gracilis*), and hog-nosed skunks (*Conepatus leuconotus*) to determine the diversity of piroplasm species that occur in these hosts. In addition, we also wanted to evaluate whether any geographic genetic variation in eastern and western samples occurs, similar to what was reported for raccoon piroplasms [10].

## Materials and methods

### Ethics

The use of samples that had been previously collected and archived from animals submitted for diagnostic evaluation was reviewed and approved by the University of Georgia’s (UGA’s) IACUC (A2007 10-186, A2008-10066, A2009 12-220, A2010 09, A2010 10-186, A2013 07-003, A2014 10-018, A2018 02-010, A2018 04-001, A2020 11-010). The collection of samples from some Texas skunks was described conducted under Texas Parks and Wildlife Department Scientific Research Permit number SPR-0390-029, Angelo State University IACUC number 18-208, and Texas Tech University IACUC number 18103-12 as described in [13]. Some Texas samples were collected during a study on *Trypanosoma cruzi* and all techniques were reviewed and approved by the UGA’s IACUC (A2008-10066, A2009 03-066). For any animals captured, all methods for capture and handling adhered to guidelines for the use of wild mammals in research.

### Methods

Spleen, frozen whole blood, or blood on nobuto filter paper were opportunistically collected from four different skunk species, including striped skunks, eastern spotted skunks, western spotted skunks, and hog-nosed skunks. Dried blood on nobuto strips was collected from Texas opportunistically from the cut distal tip of the pinna for genetic testing [24]. Striped Skunks were collected from Pennsylvania, Kentucky, Georgia, Missouri, Louisiana, Texas, Kansas, and California. Western spotted skunks were collected from California and Texas. Eastern spotted skunks were collected in North Carolina, South Carolina, and Texas [24]. Hog-nosed skunks were collected only from Texas and were almost entirely vehicle-killed animals. Because samples were opportunistically collected from animals sampled for other research purposes (i.e., Texas and California samples) or from clinical cases submitted to the Southeastern Cooperative Wildlife Disease Study (SCWDS), University of Georgia (Athens, GA) for diagnostic evaluation, there were many states with low sample sizes or limited host diversity and sample collection ranged in year, due to some cases being historic samples stored at SCWDS. All samples were stored at −20 °C until testing. Genomic DNA was extracted using a commercial kit (DNEasy Blood and Tissue kit, QIAGEN, Hilden, Germany), following the manufacturer’s protocols. All samples were initially screened for piroplasm infections using a nested PCR assay targeting the 18S rRNA gene (Table 1). To obtain the cytochrome c oxidase 1 (*cox1*) gene target for phylogenetic analyses, two additional PCR assays were conducted (Table 1). A negative water control was used in DNA extraction and PCR analysis and each step was conducted in a separate, clean space to prevent and detect contamination. Amplicons were gel extracted using a QIAquick gel extraction kit (QIAGEN) and submitted to Genewiz^©^ (South Plainfield, NJ, USA) for bi-directional sequencing. Sequences were analyzed in Geneious (Version 11.5.1). Phylogenetic trees were constructed in Geneious using FastTree v2.1 which builds trees using an approximately maximum-likelihood method and a generalized time-reversible model [25]. If multiple skunk sequences were identical, one representative sequence was used for each skunk species from each state in the phylogenetic analysis. Additional sequences from GenBank were included as representative samples of each piroplasm group and *Plasmodium falciparum* was used as an outgroup. Representative unique sequences were submitted to GenBank (PP471198–PP471209).

**Table 1:** Primers used in PCR analysis for *Babesia* species in skunks.

Species	Region	Amplicon size	Primer name	Sequence (5′ 3″)	References
All *Babesia* primary	V4 of 18S	500 bp	5.1	CCTGGTTGATCCTGCCAGTAGT	[20, 28]
			B	TGATCCTTCTGCAGGTTCACCTAC	
All *Babesia* secondary			Babesia F	GTGAAACTGCGAATGGCTCA	[18]
			Babesia R	CCATGCTGAAGTATTCAAGAC	
*Babesia* sensu stricto sp.	*cox1*	1080 bp	BabcoxF	GGAAGTGGWACWGGWTGGAC	[1]
			BabcoxR	TTCGGTATTGCATGCCTTG	
*Babesia microti-*like sp.	*cox1*	600	Cox1F133	GGAGAGCTAGGTAGTAGTGGAGATAGG	[21]
			Cox1R1130	GTGGAAGTGAGCTACCACATACGCTG	

## Results

A total of 48.4% (61/126) of skunks were positive across all locations sampled using the 18S rRNA PCR. Generally, high prevalences were noted for most sites and most skunk species; however, we sampled low sample sizes at each site (Table 2). Nonetheless, positive skunks were detected in all states sampled in this study and in general, the prevalence was lowest in Texas (although Texas also had the highest number of skunks sampled for all four species).

**Table 2:** Skunk species found to be positive for *Babesia* species infections by state.

	Striped skunk (*Mephitis mephitis*)	Eastern spotted skunk (*Spilogale putorius*)	Western spotted skunk (*Spilogale gracilis*)	Hog-nosed skunk (*Conepatus leuconotus*)
Pennsylvania	57% (12/21)	N/A	N/A	N/A
Kentucky	100% (1/1)	N/A	N/A	N/A
North Carolina	N/A	100% (5/5)	N/A	N/A
South Carolina	N/A	75% (6/8)	N/A	N/A
Georgia	100% (3/3)	N/A	N/A	N/A
Missouri	100% (2/2)	N/A	N/A	N/A
Louisiana	100% (3/3)	N/A	N/A	N/A
Texas	15% (5/33)	7% (1/15)	30% (3/10)	73% (8/11)
Kansas	100% (2/2)	N/A	N/A	N/A
California	71% (5/7)	N/A	100% (5/5)	N/A
Total	46% (33/72)	43% (12/28)	53% (8/15)	72% (8/11)

**Figure 1:**
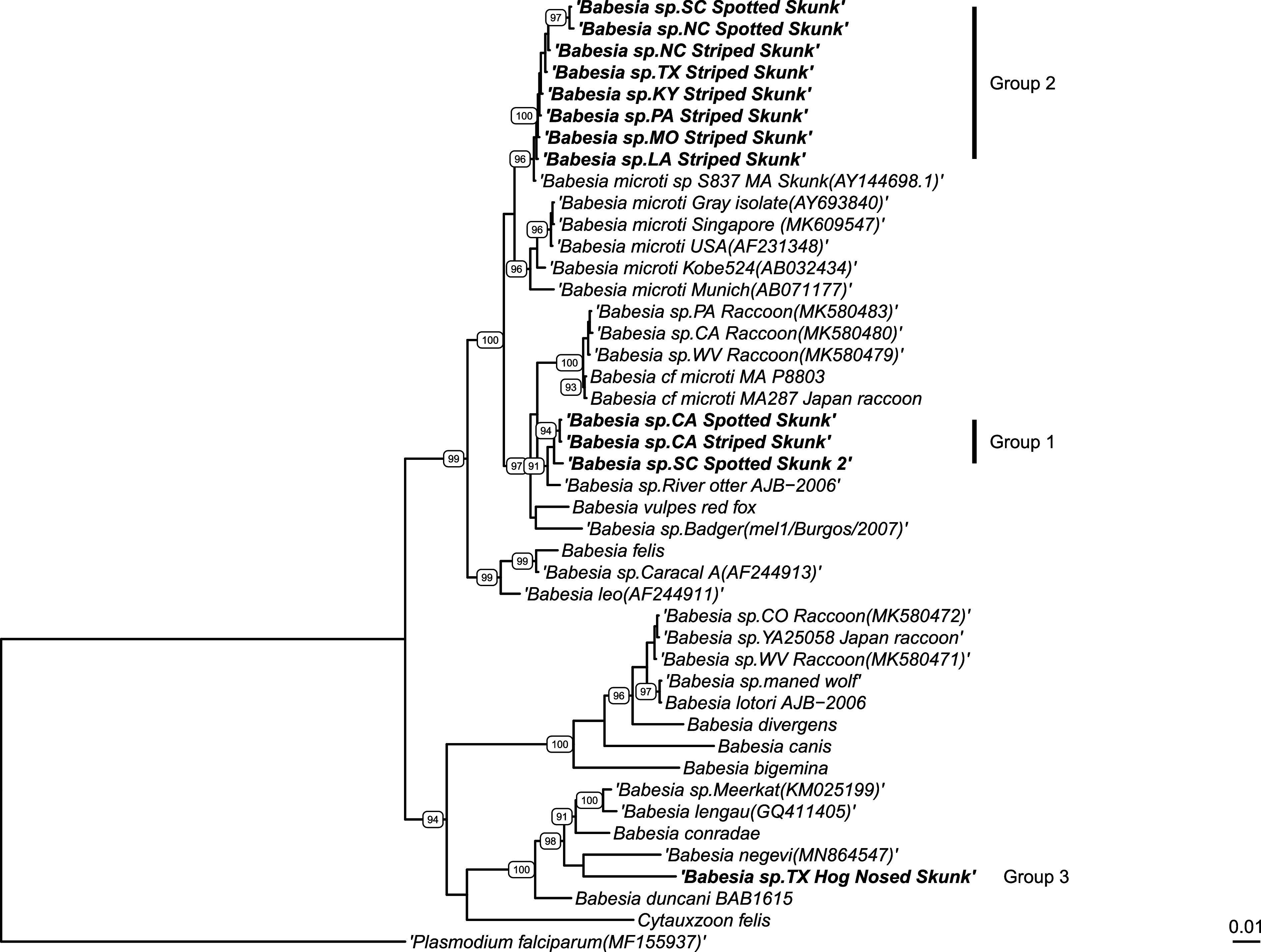
18S phylogenetic analysis using FastTree v2.1 from Geneious. Bolded samples are sequences obtained from this study. Bootstrap values above 90% are represented on the tree. *Plasmodium falciparum* was used as an outgroup.

Based on the 18S rRNA phylogenetic analysis, piroplasm sequences from our skunks grouped into three distinct clades (Fig. 1). The first group (Group 1) is a *Babesia microti-*like sp. that was most similar to the *B. microti*-like sp. in otters (95.3–98.4% similarity to sequences from otters in GenBank Supplemental Table 1), and which were both within the clade containing the *B. microti*-like species from carnivores, including *B. microti-*like sp. in raccoons and *B. vulpes*. This clade included sequences from California spotted and striped skunks, as well as a spotted skunk from South Carolina. In addition, the California sequences grouped separately from the South Carolina sequence (California samples were 100% similar but were 97.6% similar to the South Carolina sequence). The second clade (Group 2) of *Babesia* in skunks only included skunk sequences and grouped with, but separate from, *B. microti* sequences from humans and rodents (93.8–98.0% similar to *B. microti* sequences in GenBank). It is also phylogenetically distinct from the *B. microti*-like sp. of carnivores (80.6–89.0% similar). This group consists of the majority of skunk samples tested and includes spotted and striped skunks from various locations in the eastern United States. The final group (Group 3) of piroplasms in skunks is a species most similar to *B. negevi* (91.9% similar) and other species in the western piroplasms group (89.7–92.0% similar). This clade included sequences from two hog-nosed skunks from Texas.

**Figure 2:**
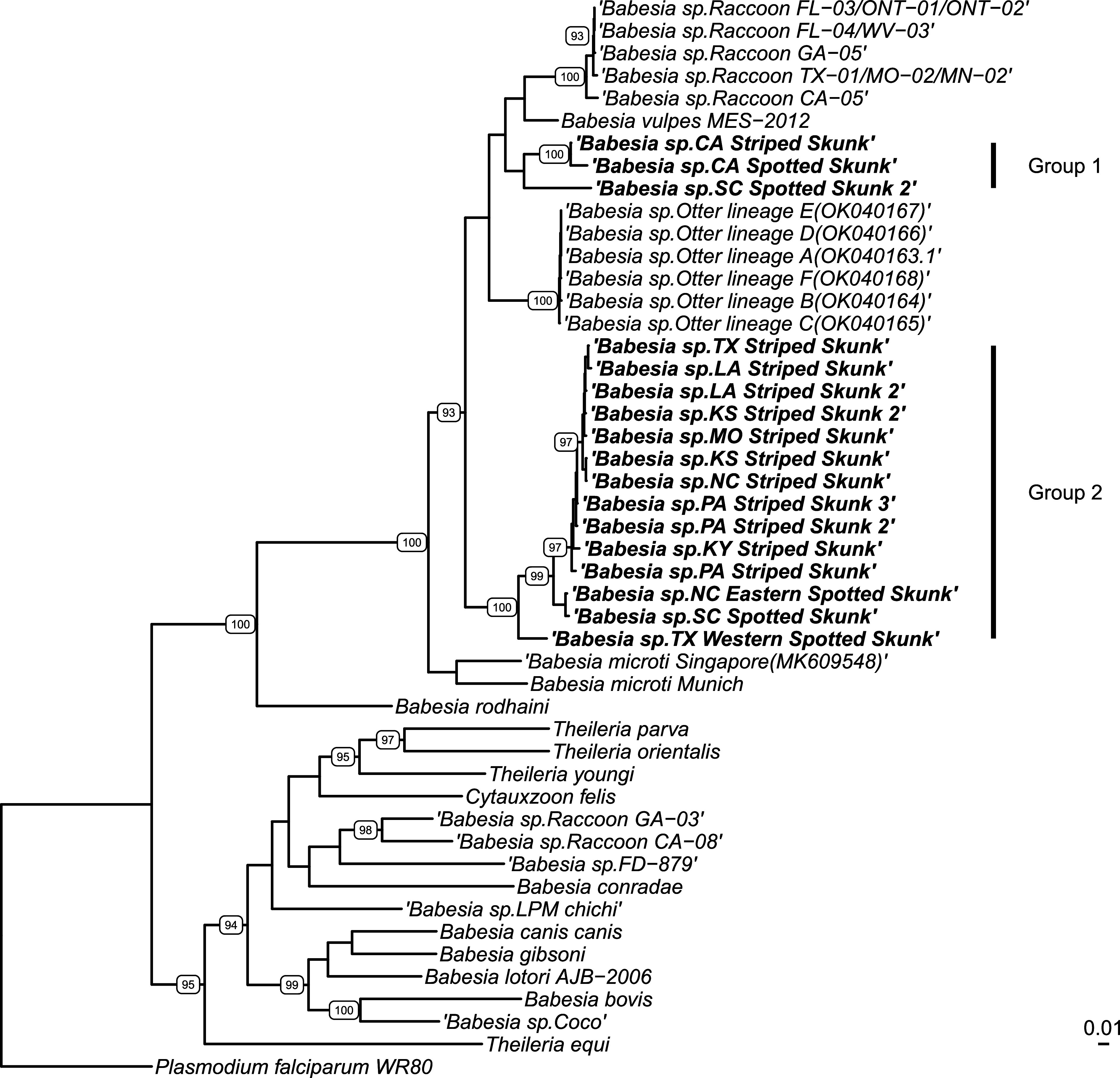
*Cox1* phylogenetic analysis using FastTree v2.1 from Geneious. Bolded samples are sequences obtained from this study. Bootstrap values above 90% are represented on the tree. *Plasmodium falciparum* was used as an outgroup.

The *cox1* gene was successfully amplified from representatives from Groups 1 and 2, but we were not able to amplify piroplasms from the two hog-nosed skunks from Texas in Group 3. Similar to the 18S rRNA gene analysis, Group 1 representatives from California skunks and the single South Carolina skunk grouped with the *B. microti*-like sp. clade from carnivores (Fig. 2). However, these samples grouped with the raccoon *B. microti*-like sp. and *B. vulpes* (87.0–91.3% similar to sequences in GenBank Supplemental Table 2) instead of with the otter *B. microti*-like sp. (87.4–88.2% similar to sequences in GenBank). There is also support in this analysis that the California skunks are more similar to each other (98.4% similar) than they are to the South Carolina sample (90.0–90.1% similar), and that the striped and spotted skunks from California are unique from each other. The *cox1* analysis also supports the data from the 18S rRNA gene for the Group 2 *B. microti*-like sp. in skunks (Group 2). In *cox1* analysis, Group 2 forms a sister clade to the *B. microti*-like sp. in carnivores (84.8–88.7% similar to sequences in GenBank) but is unique from the *B. microti* species of rodents and humans (83.7–87.1%). Within Group 2, all of the striped skunks cluster together and none of our samples from California were in this group. The spotted skunks from North Carolina and South Carolina form a distinct subclade within this group (94.1–97.1% similar to other skunks in this clade), and the Texas spotted skunk is distinct from all of these, but still within Group 2 (86.1–93.8% similar to other skunks in this clade).

Unique sequences for the 18S rRNA and *cox1* analyses were submitted to GenBank (Supplemental Table 3).

## Discussion

Three distinct clades of piroplasms were detected in skunks in this study. The overall prevalence of piroplasms in skunks in this study was 48%, which is less than a previous study where all 13 sampled skunks in Maryland were positive. However, this is not surprising as our study includes skunks from a broader geographic region which could have a varied prevalence [16]. Some of our locations sampled also had small sample sizes (as low as *n* = 1), which could bias these results.

Historically, babesiids were distinguished based on size (large vs. small piroplasms); however, phylogenetic analyses have revealed that some species that were morphologically identical are distinct species [5]. One example is a large piroplasm, *Babesia pantherae*, that was previously described in a leopard in Kenya but was never characterized at the molecular level [7], later being found based on molecular studies to be related to *B. felis,* a small babesiids in the *B. microti*-like clade [12]. Similar incongruencies have been noted with *B. gibsoni,* a small piroplasm, grouping phylogenetically with several large *Babesia* spp. and the phylogenetic placement of avian piroplasms, all of which are small and morphologically similar, in large *Babesia* clades or the smaller western piroplasms [28]. So, although *B. mephitis* was originally described from striped skunks in Maryland as a true *Babesia* species based on its large size [16], the lack of finding a true *Babesia* species in skunks since the regular use of molecular analysis suggests that *B. mephitis* is likely one of the *B. microti*-like sp. detected in this study.

Taxonomically, the piroplasms of skunks have a great deal of diversity. The *Babesia microti-*like sp. in California skunks and a South Carolina spotted skunk (Group 1) in the carnivore group is more phylogenetically similar to *Babesia microti-*like sp. in otters for the 18S analysis, and for the *cox1* analysis is more similar to *B. vulpes* and *Babesia microti-*like species infecting raccoons. The second group we found in skunks matches the piroplasm of a skunk found in a previous study in Massachusetts and is phylogenetically uncertain in its placement within the *B. microti* complex [13]. In our study, this skunk group was more similar to *B. microti* of rodents and humans in the 18S analysis, and the *cox1* analysis showed it to be more similar to the *B. microti*-like sp. of carnivores. Regardless of the analysis, the piroplasms in this skunk group all form their own clade, further supporting the hypothesis that there is likely a skunk-specific group in the *B. microti* complex of piroplasms [14]. This further highlights the importance of having multiple gene targets when analyzing the piroplasms that occur in the *B. microti* and *B. microti*-like clade in order to not misidentify species within this group.

The final group of piroplasms identified in hog-nosed skunks was phylogenetically most similar to *Babesia negevi* and other western piroplasms, such as *B. conradae* and *B. duncani*. To our knowledge, this is the first finding of a western piroplasm in any skunk species. We were only able to acquire 18S rRNA gene sequence for this group. In our previous study on piroplasms with raccoons, we were also unable to amplify piroplasms in the western piroplasms group, and this inability to amplify *cox1* may be due to the lack of primer binding for some of the species in the western piroplasms group [10]. The natural range of the hog-nosed skunks in the United States includes southwestern states such as Texas, New Mexico, and Arizona and the finding of a piroplasm in the western piroplasms group in hog-nosed skunks highlights the need for further research on piroplasms in this wildlife species [17].

Many *Babesia* sp. infections in wildlife result in little to no clinical disease. However, occasionally when co-infected with another pathogen, *Babesia* infections can cause clinical disease [22, 23]. Clinical infections with *B. microti-*like species in carnivores can also occur when in a non-natural host. For example, *B. vulpes* infections in domestic dogs can cause thrombocytopenia and hemolytic anemia [4]. Additionally, an otter infected with a *B. microti*-like sp. presented with lethargy, anemia, and anorexia, eventually leading to death [11]. Finally, infections in young animals may lead to disease that is unapparent, such as young raccoons infected with *B. lotori* experiencing splenomegaly [9]. It is unknown to what extent wildlife species may experience disease with certain piroplasm parasites, as often animals that are being tested have either been submitted from presumed healthy animals from the wild or those submitted to rehabilitation or diagnostic facilities that may have other issues. In the case of the skunks submitted for this study, none were submitted due to suspected piroplasm infection. However, full health assessments on all animals were not completed and therefore some clinical disease may have been unnoticed. Further research is needed on the clinical relevance of piroplasm infections for many wildlife species, particularly threatened or endangered species.

Based on our previous study of piroplasms in raccoons and other studies on *B. microti*-like sp. piroplasms, we expected to see geographic genetic variation in the skunk piroplasms [10, 19]. We did see some evidence of this, with Group 1 containing distinct clusters for the California samples and the South Carolina sample. However, because no other samples in this group were detected in our study, we are unable to determine if this is due to true geographic genetic variation in the species. For Group 2 in the 18S analysis, there seems to be no clear difference in geography. However, most of the samples are from eastern states with the westernmost sample being a Texas striped skunk. For the *cox1* analysis, all the striped skunk samples cluster into one large group with no evidence of geographic variation. Within this large piroplasms of skunks group, there is a sister clade that includes a North Carolina and a South Carolina spotted skunk, providing support for the western spotted skunk samples being distinctly different. This gives some evidence for geographic variation in the spotted skunk samples; however, more representatives from skunks from western states are needed to determine if there is any geographic variation.

In conclusion, skunks were infected with at least three different *Babesia* species in the United States, including two species in the *Babesia microti* complex and one species in the western piroplasms group that was only found in hog-nosed skunks from Texas. This study was a small, opportunistic survey of skunks from various locations throughout the United States, but it highlights the need for further research to better understand piroplasms in skunks. Paired blood smears and further molecular analysis of the different piroplasm species in skunks are needed in order to best characterize and describe these species.

## Supplementary material

The supplementary material for this article can be found at https://www.parasite-journal.org/10.1051/parasite/2024043/olm. *Supplemental Table 1*: Percentages of identity of 18S sequences. *Supplemental Table 2*: Percentages of identity of *cox1* sequences. *Supplemental Table 3*: Submitted GenBank accession numbers for skunk 18S rRNA and cox1 phylogenetic analyses.
