# Supplementary material for: Diversity of Babesia spp. in skunks from selected states in the United States of America
Source: Parasite. 2024 Jul 24;31:42. doi: 10.1051/parasite/2024043 (PMC11271706; doi:10.1051/parasite/2024043)
Supplement: Supplementary file 2 — Supplemental Table 3: Submitted GenBank accession numbers for skunk 18S rRNA and cox1 phylogenetic analyses. [file parasite-31-42-s2.pdf]

Supplemental Table 3: Submitted GenBank accession numbers for skunk 18S rRNA and *cox1*

Sample ID	Gene Target	GenBank Accession Number
CA_Spotted_Skunk	18S rRNA	PP471198
CA_Striped_Skunk	18S rRNA	PP471199
SC_Spotted_Skunk	18S rRNA	PP471200
KY_Striped_Skunk	18S rRNA	PP471201
LA_Striped_Skunk	18S rRNA	PP471202
MO_Striped_Skunk	18S rRNA	PP471203
PA_Striped_Skunk	18S rRNA	PP471204
NC_Spotted_Skunk	18S rRNA	PP471205
NC_Striped_Skunk	18S rRNA	PP471206
TX_Hog_Nosed_Skunk	18S rRNA	PP471207
TX_Striped_Skunk	18S rRNA	PP471208
SC_Spotted_Skunk2	18S rRNA	PP471209
SC_Spotted_Skunk	*cox1*	PP623016
SC_Spotted_Skunk2	*cox1*	PP623017
KY_Striped_Skunk	*cox1*	PP623018
MO_Striped_Skunk	*cox1*	PP623019
LA_Striped_Skunk	*cox1*	PP623020
LA_Striped_Skunk2	*cox1*	PP623021
CA_Spotted_Skunk	*cox1*	PP623022
NC_Striped_Skunk	*cox1*	PP623023
NC_Eastern_Spotted_Skunk	*cox1*	PP623024
KS_Striped_Skunk2	*cox1*	PP623025
KS_Striped_Skunk	*cox1*	PP623026
TX_Striped_Skunk	*cox1*	PP623027
TX_Western_Spotted_Skunk	*cox1*	PP623028
PA_Striped_Skunk	*cox1*	PP623029
PA_Striped_Skunk2	*cox1*	PP623030
PA_Striped_Skunk3	*cox1*	PP623031
CA_Striped_Skunk	*cox1*	PP623032

phylogenetic analyses.
